# Biosynthesis of magnetic nanoparticles by human mesenchymal stem cells following transfection with the magnetotactic bacterial gene *mms6*

**DOI:** 10.1038/srep39755

**Published:** 2017-01-04

**Authors:** Alistair Elfick, Grigore Rischitor, Rabah Mouras, Asim Azfer, Lisa Lungaro, Marc Uhlarz, Thomas Herrmannsdörfer, John Lucocq, Wesam Gamal, Pierre Bagnaninchi, Scott Semple, Donald M Salter

**Affiliations:** 1 University of Edinburgh, Institute for Bioengineering, School of Engineering, Edinburgh, EH9 3FB, UK; 2 University of Edinburgh, UK Centre for Mammalian Synthetic Biology, Edinburgh, EH9 3FB, UK; 3 University of Edinburgh, Centre for Genomics and Experimental Medicine, MRC IGMM, Edinburgh, EH4 2XU, UK; 4 Helmholtz-Zentrum Dresden-Rossendorf, Dresden High Magnetic Field Laboratory (HLD-EMFL), Dresden, 01328, Germany; 5 University of St Andrews, School of Medicine, St Andrews, KY16 9TF, UK; 6 University of Edinburgh, Centre for Regenerative Medicine, Edinburgh, EH16 4UU, UK; 7 University of Edinburgh, Centre for Cardiovascular Science, Edinburgh, EH16 4TJ UK

## Abstract

The use of stem cells to support tissue repair is facilitated by loading of the therapeutic cells with magnetic nanoparticles (MNPs) enabling magnetic tracking and targeting. Current methods for magnetizing cells use artificial MNPs and have disadvantages of variable uptake, cellular cytotoxicity and loss of nanoparticles on cell division. Here we demonstrate a transgenic approach to magnetize human mesenchymal stem cells (MSCs). MSCs are genetically modified by transfection with the *mms6* gene derived from *Magnetospirillum magneticum* AMB-1, a magnetotactic bacterium that synthesises single-magnetic domain crystals which are incorporated into magnetosomes. Following transfection of MSCs with the *mms6* gene there is bio-assimilated synthesis of intracytoplasmic magnetic nanoparticles which can be imaged by MR and which have no deleterious effects on cell proliferation, migration or differentiation. The assimilation of magnetic nanoparticle synthesis into mammalian cells creates a real and compelling, cytocompatible, alternative to exogenous administration of MNPs.

MSCs have wide therapeutic potential for tissue repair and in cancer therapy but problems relating to targeting, engraftment and localisation persist. Labelling of MSCs with superparamagnetic iron oxide nanoparticles allows *in vivo* tracking by MR imaging and the potential to target cells to sites of injury or surgical implants to facilitate tissue repair^1^,^2^,^3^,^4^. Identifying the optimal method to magnetize MSCs has proven challenging. Unlike phagocytic cells, such as macrophages, MSCs have a relatively poor intrinsic capacity to ingest extrinsically applied MNPs, although this may be improved by the use of gene transfection agents^5^ or coating of MNPs with a range of substances including starch, poly aspartic acid and dextran sulphate^6^. However, uptake of MNPs by MSCs is variable over a population and may have detrimental effects on cell proliferation and migration^7^. Furthermore, the efficacy of MSC imaging, targeting and retention may be compromised by dilution of MNP content in progeny following proliferation and the possibility of MNP release and uptake by alternate cells. An alternative approach to magnetise MSCs would be to promote *de novo* synthesis of intracellular MNPs as occurs naturally in magnetotactic bacteria^8^,^9^. These bacteria contain a unique intracellular organelle, the magnetosome, which comprises a magnetic nanoparticle, typically magnetite (Fe_3_O_4_) surrounded by a lipid bilayer membrane^10^,^11^. Magnetosome formation is dependent on a conserved region within magnetotactic bacterial DNA called the magnetosome island (MAI) comprising *mamAB, mamGFDC, mms6* and *mamXY* operons^12^. The *mms6* operon comprises the genes *mgr4074, mms6, mmsF, mms36* and *mms48* and their contribution to magnetosome biogenesis is beginning to be understood^13^. Mms6 promotes the formation of uniform isomorphic superparamagnetic magnetite nano-crystals and helps regulate the crystal morphology of magnetite^14^. Significantly, recombinant Mms6 binds iron and aids formation of magnetite particles *in vitro* that are similar to those of magnetosomes^13^. Here we show that transfection of human MSCs with the magnetobacterial gene *mms6* is sufficient to allow *de novo* synthesis of intracellular magnetic nanoparticles without functional detriment facilitating theragnostic applications of magnetic MSCs.

## Results

### *mms6* expression and nanoparticle formation in human MSCs

**Figure 1:**
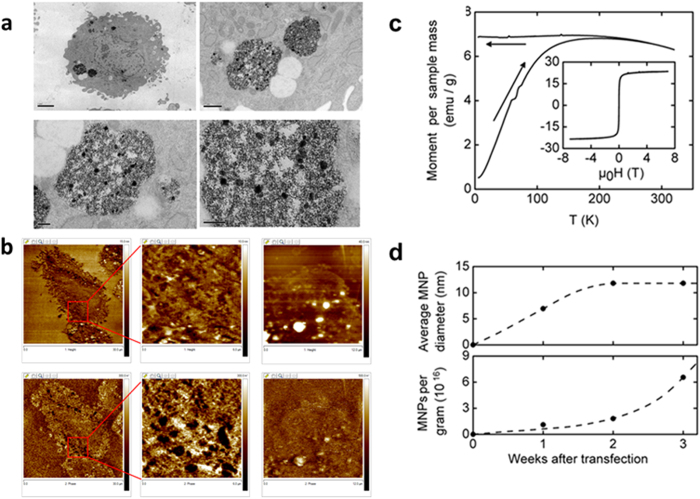
Production of magnetic nanparticles by *mms6* gene transfected human MSCs cultured in the presence of 34 mM ferric quinate. (**a**) TEM: scale bars upper left 2 μm, upper right 0.5 μm, lower left 0.2 μm, lower right 0.2 μm. (**b**) AFM/MFM of *mms6* transfected MSCs (left and central panels) and untransfected MSCs (right panel). The upper row demonstrates AFM topographic images whilst the lower demonstrates the equivalent MFM images. Magnetic particles are identified in the MFM images of as clusters of black spots due to attractive forces between the magnetised tip and the nanoparticles. Image widths: left panels 30 mm, central panels 6 mm, right panels 12 mm. (**c**) SQUID magnetometry. A. Magnetization of MSCs after the third week after *mms6* transfection. Main panel: Temperature dependence of the magnetosome contribution to the magnetic moment after cooling in zero field and subsequent heating (lower leg) and cooling (upper leg) in μ_0_H = 0.01T, related to dry sample mass. Inset: Magnetic field dependence of the mass-specific magnetic moment for the same sample at body temperature. (**d**) Upper panel. Estimate of nanoparticle diameter based on the assumption that they consist of superparamagnetic Fe_3_O_4_ particles. Lower panel: estimate of nanoparticle number per gram of dry sample material, derived from estimated diameter and saturated magnetic moment.

The *Magnetospirillum magneticum* AMB-1 *mms6* DNA bacterial sequence was codon optimized for mammalian expression and a Kozak sequence added then synthesized. The optimized sequence was synthesised by MRGene GmbH, Germany and cloned in their proprietary vector with SacI and KpnI restriction sites. The synthetic *mms6* gene was cloned into a pcDNA3.1 expression vector and transfected into human adipocyte derived MSCs with either X-tremeGENE HP or FugeneHD. Cells were cultured in the presence of 34 mM ferric quinate which is frequently used as a source of iron for magnetobacterial culture and magnetosome formation^15^. Expression of the *mms6* gene in the transfected cells at 10, 15 and 21 days post-transfection was confirmed by RT-PCR (Supplementary Fig. 1). Sanger DNA sequencing of the PCR transcripts of the *mms6* gene 10, 15 and 21 days post-transfection showed 100% identity with the inserted gene (Supplementary Fig. 2). Transfected cells cultured in the presence of 34 mM ferric quinate, after 10–14 days, had a distinct dark golden yellow appearance under light microscopy. By transmission electron microscopy (TEM) nanoparticles are identified within vacuoles, up to 1 μm diameter, in the cytoplasm of the *mms6* transfected cells at 1, 2 and 3 weeks in culture (Fig. 1a). Unlike the highly ordered cubo-octahedral crystals of magnetite crystals of *Magnetospirillum magneticum* AMB-1 in culture^16^, or those synthesised by partial oxidation of ferrous hydroxide in the presence of recombinant magnetotactic bacterial protein Mms6 *in vitro*^17^, the nanoparticles produced by the *mms6* transfected MSCs appeared unstructured and ranged from 10 to 500 nm in size although it is not clear whether the larger nanoparticles are aggregates of the smaller particles. TEM images of untransfected MSCs in which no nanoparticles are identified and untransfected MSCs loaded with FluidMag DXS magnetic nanoparticles in which intracytoplasmic nano sized particles are present, are shown in Supplementary Fig. 3.

### Intracellular nanoparticles demonstrate magnetic properties and generate MR image contrast

Atomic force microscopy/magnetic force microscopy (AFM/MFM) indicated that the synthesised nanoparticles were magnetic. AFM/MFM studies were undertaken on FeCl_3_ powder used for ferric quinate formation in the cell culture medium, FluidMag DXS magnetic nanoparticles, MSCs loaded with FluidMag DXS magnetic nanoparticles and both *mms6* expressing and untransfected MSCs. AFM/MFM studies with FeCl_3_ powder and FluidMag DXS magnetic nanoparticles dispersed on a glass cover slip demonstrate that the FeCl_3_ powder has no magnetic properties and that both dispersed cell free and intracellular FluidMag DXS magnetic particles could be detected by AFM/MFM (Supplementary Fig. 4). For AFM/MFM microscopy on intact *mms6* transfected and untransfected MSCs 80 nm thick sections where placed on glass cover slips. Obtained data are shown in Fig. 1b. The topographic images (upper row) show the morphological variation of the surface with cells clearly identified. The MFM images (bottom row) show the presence of magnetic structures in the transfected cells, in the addition to some morphologic artefact. These features are mainly present in relation to the cell cytoplasm consistent with the TEM observations. No magnetic structures were identified in the untransfected MSCs cultured with ferric quinate. Superconducting quantum interference device (SQUID) magnetometry was used to further confirm the magnetic nature of the nanoparticles synthesised by the *mms6* transfected cells. Two different measurement modes were used: First, we measured the temperature-dependent magnetization in a small, fixed field (μ_0_H = 0.01 T) between body temperature, *T* = 310 K, and *T* = 5 K. Second, we measured the field dependence of the magnetization at human body temperature in magnetic fields up to μ_0_H = 7 T. Results of these measurements for MSCs at the third week after transfection are shown in Fig. 1c and d. In the main panel of Fig. 1c, the magnetic moment per dry-sample mass is shown after cooling in zero field to *T* = 5 K, then applying a small field of μ_0_H = 0.01 T, and subsequently heating (lower leg of the curve) and cooling (upper leg of the curve) in this field. The inset to Fig. 1c shows the field dependence of the mass-specific magnetic moment for the same sample of MSCs after the third week after transfection, taken at body temperature. In combination with a high value of saturated moment (μ_mass_ = 23.5 emu/g), the low coercive field of *μ*_0_*H*_c_ = 2×10^−3^ T confirms the superparamagnetic character of the nanoparticles. A temperature scaling of these features is expected for nanoparticles of the same material (e.g., Fe_3_O_4_) and same shape, but of different average diameters, with the blocking temperature, *T*_*B*_, increasing linearly with the average volume of the superparamagnetic particles^18^. As a measure of *T*_*B*_ we took the intersection temperature between field-heated and field-cooled curves. Comparison with a sample of MSCs which were treated with commercially available FluidMag DXS Fe_3_O_4_ nanoparticles of 100 nm diameter (not shown) enabled estimation of the average diameter of the nanoparticles in transfected cells following one, two and three weeks in culture (Fig. 1d upper panel). The size of the magnetic particles is predicted to plateau at an average value of 12 nm after the second week. The saturated moment values for each sample, together with the estimated average diameter of the nanoparticles, allows estimation of the nanoparticle number after the first, second and third week post transfection. Contrary to the stalling of average nanoparticle diameter by week three there appeared to be continued production and accumulation of nanoparticles (Fig. 1d lower panel) supporting TEM observations. SQUID magnetometry of untransfected cells under identical culture conditions showed no magnetic behaviour (results not shown).

Currently, superparamagnetic iron-oxide nanoparticles (SPIONs) are being used to magnetically label stem cells to allow *in vivo* imaging by MR and to potentially aid localisation and retention of the loaded cells at the site of interest^19^. To assess whether the *mms6* transfected, MNP-bearing MSCs could be identified by MR, an *in vitro* phantom study was undertaken. Non-transfected MSCs, *mms6* transfected MSCs and MSCs loaded with commercially available FluidMag DXS MNPs were placed in 1% agarose gel in 50 ml falcon tubes and subjected to MR imaging (Supplementary Fig. 5). The T_2_* values (ms) of the empty phantom and the phantom containing untransfected cells were 89.6 + 3.5 and 73.7 + 4.2 respectively. The T_2_* values of the *mms6* transfected cells ranged from 24.9 + 6.8 to 39.2 + 5.9 whereas that of the commercial MNP loaded MSCs was 20.2 + 2.5 confirming their potential for *in vivo* tracking.

### *mms6* transfected, nanoparticle containing MSCs maintain cell proliferation, migration and differentiation

**Figure 2:**
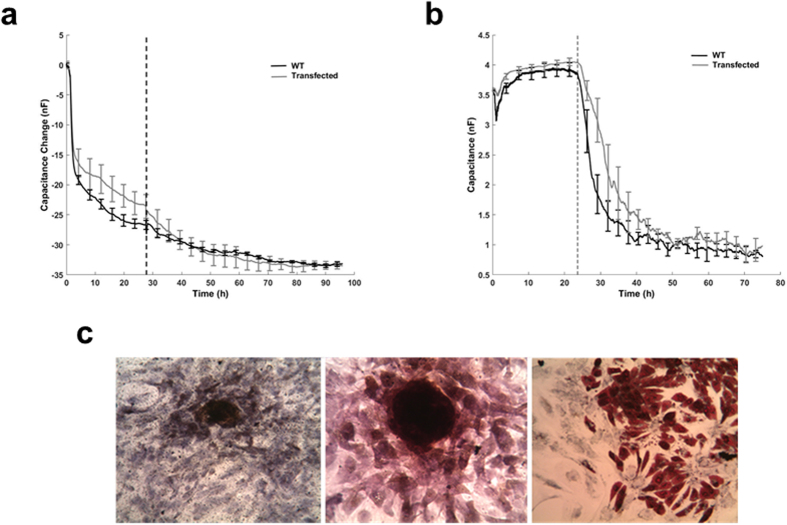
Effect of intrinsic magnetic nanoparticle production on *mms6* transfected MSC proliferation, migration and lineage differentiation. (**a**) Electric cell-substrate impedance sensing (ECIS) Assay. Proliferation of wild type (WT) and *mms6* transfected MSCs over the course of 4 days. (**b**) Electric cell-substrate impedance sensing (ECIS) Assay. Capacitance measured in a medusa array demonstrating cell migration. (**c**) Histochemical demonstration of specific lineage differentiation of mms6 transfected MSCs. Left panel – von Kossa stain for calcium deposition in cells cultured in osteogenic medium; middle panel-safranin O staining of cells cultured in chondrogenic medium; right panel-oil-red O staining of cells cultured in adipogenic medium.

To assess whether *mms6* transfection and assimilation of magnetic nanoparticle synthesis might have adverse effects on MSC function and their ability to aid specialised tissue repair, studies on cell proliferation, migration and differentiation were undertaken (Fig. 2a and b). The proliferative capacity of the MSCs was investigated with electric cell-substrate impedance sensing (ECIS) to investigate whether *mms6* transfected MSCs differed from wild-type. The forty gold microelectrodes per well of the ECIS instrument enabled the quantification of cell coverage through impedance measurement in real-time (every 180s) over 4 consecutive days. No difference in the time-course cell coverage (Fig. 2a) was seen between transfected MSCs and controls. Subsequently, a medusa array was used with two addressable 250 μm gold microelectrodes per well to study cell migration (Fig. 2b). An electric fence was applied to one of the two electrodes while on the control electrode both transfected and wild-type cells, plated at high density, attached and spread on the single electrode with the same rapid kinetics until they reached a plateau at T_1_. On the second electrode, the electric fence was then turned off at T_1_, allowing cells to migrate into the cell-free area and resulting in a decrease in capacitance. Migration rates^20^ were calculated (n = 3) with no statistical difference (p > 0.05) observed between transfected MSCs and wild-type control cells. Importantly, *mms6* transfected MSCs also remain pluripotential when cultured in osteogenic, chondrogenic or adipogenic media as demonstrated by RT-PCR (not shown) and histochemical staining (Fig. 2C). *mms6* transfected MSCs cultured for 3 weeks in osteogenic media expressed *COL1A1* and *BGLAP* and stained positively with von Kossa stain indicating calcium deposition. Those cells cultured in chondrogenic expressed *COL2A1* and *ACAN* and stained positively with alcian blue, whilst culture in adipogenic media resulted in expression of *PPARG* and oil-red O staining indicating that they maintained the capacity for specific lineage differentiation.

## Discussion

Cell-based therapy is an emerging area of regenerative medicine in which stem or progenitor cells are delivered either locally or systemically, principally by the blood stream. Major obstacles in the field, including retention of cells at a particular site and identifying the fate of locally or systemically injected cells^21^,^22^, are being challenged by loading of cells magnetic nanoparticles^23^,^24^,^25^. Currently, SPIONs are being used to magnetically label stem cells to allow *in vivo* imaging by MR and to potentially aid localisation and retention of the loaded cells at the site of interest^19^. In the current study we have demonstrated the utility of a transgenic approach using Magnetospirillum magneticum AMB-1 *mms6* DNA bacterial sequence for magnetising MSCs without adverse effects on cell proliferation, migration and differentiation.

Previous studies have demonstrated that stable transfection of mouse and human cancer cell lines with *MagA*, which encodes for a magnetotactic bacterial protein, results in increased iron labelling of the cells allowing MR imaging^26^,^27^. A study in which *MagA* was transfected into a human embryonal kidney cell line demonstrated intracellular production of 3–5 nm diameter nanoparticles which, following magnetic separation, were seen to consist primarily of magnetite^28^. To assess whether another magnetobacterial gene *mmsF* had similar properties to that of *mms6* in our system we have recently transfected human embryonic bone marrow derived MSCs with a codon-optimized *mmsF* gene and cultured the cells under similar conditions to that of the *mms6* transfected cells described in the current study. TEM demonstrated production of intracellular nanoparticles which were shown to have magnetic properties by SQUID magnetometry (Supplementary Figs 6 and 7). Thus it would appear that transfection of mammalian cells by a number of different MTB genes may facilitate production of magnetic nanoparticles. MagA is a putative iron transporter and provides an increase in total cellular iron in response to an extracellular iron supplement^27^. The mechanism by which *mms6* expression results in production of intracellular magnetic nanoparticles in MSCs is not yet understood. Recombinant Mms6 protein has previously been shown to have iron binding activity, facilitating generation of uniform magnetic crystals by co-precipitation of ferrous and ferric ions^29^. Furthermore, work by Matsunaga and colleagues indicates that, because of a high affinity for iron ions and a highly charged electrostatic quality, Mms6 protein acts as a template for nucleus formation and/or a regulator of crystal size and morphology^17^,^30^,^31^. It has been proposed recently that Mms6 may self-assemble as a protein raft to display regular binding sites for iron ions to nucleate magnetite formation^32^. Interestingly, MmsF, encoded within the *mms6* gene cluster, is also responsible for defining crystal size and morphology and may regulate magnetite crystal formation through similar mechanisms to Mms6^33^.

Genetically modified MSCs expressing magnetic nanoparticles have considerable potential in regenerative medicine, both for MR imaging and for magnetic targeting by application of a magnetic field over the area of interest to attract and retain the labelled cells after either local or systemic injection. Typically this may be with an external magnet^34^ but increasingly magnetic implants are being developed, as in the orthopaedic field where insertion of magnetic scaffolds with or without permanent magnets can be used to aid healing of massive bone defects and chondro-osseous defects^35^,^36^,^37^, or in vascular surgery where magnetic stents can aid targeting of magnetised endothelial cells^38^. The assimilation of magnetic nanoparticle synthesis into mammalian cells creates a real and compelling, cytocompatible, alternative to exogenous administration of SPIONs. Importantly, any progeny of the cells could also synthesise their own nanoparticles, overcoming a significant practical constraint for SPION use. This motivates future technical development in stabilisation of expression through chromosomal integration.

## Methods

### Expression of *mms6* in mammalian cells

*Magnetospirillum magneticum* AMB-1 *mms6* DNA bacterial sequence was codon optimized for mammalian expression and a Kozak sequence added then synthesized. The optimized sequence was synthesised by MRGene GmbH, Germany and cloned in their proprietary vector with SacI and KpnI restriction sites. The synthetic *mms6* gene was then cloned into pcDNA3.1 expression vector (ThermoFisher Scientific) at BamH1 and EcoRI restriction sites. Transfection into the human adipose derived stem cells (STEMPRO^®^ - Human Adipose-Derived Stem Cell Kit Human, Invitrogen) was carried out with either X-tremeGENE HP and FugeneHD transfection reagents (Roche Scientific) in accordance with manufacturer protocols.

### *mms6* gene expression

Total cellular RNA was extracted from cells by using the RNeasy Kit (Qiagen. Hilden. Germany). Prior to reverse transcription 1 μg of RNA was treated with DNAse-I amplification grade (Sigma, USA). The cDNA synthesis was performed by treating the RNA with Oligo(dT) primers (Epicentre, USA, MMLV High Performance Reverse Transcriptase). After cDNA synthesis the solution was diluted with distilled water to a final volume of 200 μl. A 2μ1 aliquot of the cDNA product was amplified as follows: Denaturation at 95 °C for 3 min, 35 cycles of amplification associated with denaturation at 95 °C for 15 s, annealing at 60 °C and extension at 72 °C for 1 min. The *mms6* primers used were (1) internal primer pair for initial gene expression studies-forward: 5′- GTGGGGCCCGATTATTCT-3′; reverse: 5′-TCACGCAGTTCCACTTCTTC-3′, the amplified PCR product of 108 bp was resolved in 2.5% agarose gel and (2) for gene expression and DNA sequencing – forward: 5′-ATGGGCGAAATGGAACGTGAAG-3′; reverse 5′-TCATGGTGGCGCCAGCGCATC-3′.

### Cell Culture

Transfected human adipose-derived stem cells were cultured according to the manufacturer’s instructions in MesenPRO RS™ Medium in the absence, or presence, of ferric quinate at a final concentration of 34 mM. For assessment of specific lineage differentiation capacity *mms6* transfected MSCs were cultured to confluence, after which the medium was replaced with specific differentiation media including StemPro^®^ Osteogenesis Differentiation media (Gibco), StemPro^®^ Chondrogenesis Differentiation medium (Gibco) and StemPro^®^ Adipogenesis Differentiation medium (Gibco) according to the manufacturer’s instructions. The MG63 osteosarcoma cell line was used in initial gene transfection and expression studies (see Supplementary Fig. 1).

### Transmission Electron Microscopy

Following monolayer culture cells were detached, fixed in 3% glutaraldehyde in 0.1 M sodium cacodylate buffer, pH 7.3, for 2 hours then washed in three 10 minute changes of 0.1 M sodium cacodylate and post-fixed in 1% osmium tetroxide in 0.1 M sodium cacodylate for 45 minutes before an additional three 10 minute changes of 0.1 M sodium cacodylate buffer. The samples were then dehydrated in 50%, 70%, 90% and 100% normal grade acetone for 10 minutes each followed by two 10-minute changes in analar acetone before being embedded in Araldite resin. Sections were cut on a Reichert OMU4 ultramicrotome (Leica Microsystems (UK) Ltd, Milton Keynes), stained with Toluidine Blue and viewed in a light microscope to select suitable areas for investigation. Ultrathin sections, 60 nm thick, were cut from selected areas, stained in uranyl acetate and lead citrate then viewed in a Phillips CM120 transmission electron microscope (FEI UK Ltd, Cambridge, England). Images were taken on a Gatan Orius CCD camera (Gatan UK, Oxon, England).

### Atomic Force Microscopy (AFM) and Magnetic Force Microscopy (MFM)

AFM-MFM imaging was performed under ambient conditions with a commercial scanning probe microscope, Bioscope II (Bruker-Nano) using magnetic probes (MESP-RC cantilevers) that were properly magnetised just before their use. Untransfected and transfected cells were embedded in Araldite resin, and 80 nm thick sections were cut on a Reichert OMU4 ultramicrotome (Leica Microsystems (UK) Ltd, Milton Keynes) and placed on glass coverslips for AFM-MFM imaging. To obtain simultaneous topographic and magnetic images the LiftMode technique was used. First, the tip is scanned over the surface of the sample to record obtain the topographic information using the taping mode. Then the tip is raised just above the sample surface by an offset (20 nm) called lift height. The surface topography from the initial scan is added to the lift height to maintain constant separation during the lifted scan. Magnetic interactions are detected during this second pass and information about the magnetic field above a sample are gathered. Using the LiftMode technique, topographical features are virtually absent from the MFM images.

### Magnetic Property Analysis

Magnetization measurements were performed with a superconducting quantum interference device (SQUID) magnetometer. This instrument measures the total magnetic moment of a sample, including all atomic and molecular magnetic contributions. Magnetization data were taken at temperatures 5 K < T < 350 K using a liquid-He cooled variable-temperature insert installed in the commercial SQUID-magnetometer apparatus (MPMS, Quantum Design Inc., San Diego, USA).

Samples were centrifuged for 5 minutes at 5000 g and the supernatant fluid was decanted. The remaining cell material was dried carefully for 12 hours at room temperature. Then, the sample material was weighed using a Sartorius Micro M3P balance to derive mass-specific values, transferred to the sample holder, and measured at respective fields and temperatures. Initially, the properties of a control sample of transfected MSCs grown in non-iron containing media were determined. This diamagnetic contribution of the pure-protein component to magnetization was later subtracted from the measured properties of the other sample materials to obtain the magnetization component originating from the nanoparticles. Two different measurement modes were used for all samples: First, we measured the temperature-dependent magnetization in a small, fixed field (μ_0_H = 0.01T) between body temperature, T = 310 K, and T = 5 K. Second, we measured the field dependence of the magnetization at body temperature in magnetic fields up to μ_0_H = 7T. During the measurements, the magnetic field, generated by a superconducting coil, was either held constant while measuring at varying temperatures, or was swept at constant temperature, while the samples were consistently moved through a pick-up coil system connected to the SQUID via a flux transformer.

### MR Imaging

MSCs were transfected described above and cultured for three weeks, harvested and fixed in 1:1 methanol:acetone for 5 minutes and loaded in 1% agarose gel in 50 ml falcon tubes. Magnetic resonance was performed using the head matrix coil of a 3 T MRI system (MAGNETOM Verio, Siemens AG, Healthcare Sector, Erlangen, Germany). Phantoms were suspended in a water bath to reduce artefact. Multi-gradient-echo T2* acquisitions were acquired from 2.7–21.3 ms and used to generate T_2_* values (ms) using a standard least-squares fitting routine in Matlab (Mathworks).

### Electric cell-substrate impedance sensing (ECIS) Assay

Impedance characterisation of transfected cells behaviour was conducted with the ECIS system (ECIS Zθ, Applied Biophysics, NY, USA). In brief, cells were grown on eight-well ECIS arrays (8W10+; Applied Biophysics, NY, USA) containing forty 250-μm gold microelectrodes per well. Measurements were performed directly in cell culture medium, allowing real-time monitoring. Both the ECIS arrays and the measurement station were kept in an incubator with high humidity at 37 °C and 5% CO_2_. Wild type and transfected cells were plated at low concentration in three wells each (N = 3) at t = 0, and the impedance was measured against time in an incubator with a sampling frequency of 1/180s for several days. Impedance of a cell-free electrode in media was also measured as a background signal. For the Medusa array studies two independently addressable 250 μm diameter gold microelectrodes per well were used. One of the electrodes was submitted to a high electric field “fence” (40 kHz, 1 mA, 1s, 5 cycles per min) to prevent cell attachment, while cells were free to attach on the second one. When the so-called electric fence was lifted cell migration occurred to repopulate the cell-free electrode. Matlab was then used to perform one-way ANOVA and Tukey–Kramer multicomparison tests to determine whether the groups under investigation were significantly different from each other.

## Additional Information

**How to cite this article**: Elfick, A. *et al*. Biosynthesis of magnetic nanoparticles by human mesenchymal stem cells following transfection with the magnetotactic bacterial gene *mms6. Sci. Rep.* **7**, 39755; doi: 10.1038/srep39755 (2017).

**Publisher's note:** Springer Nature remains neutral with regard to jurisdictional claims in published maps and institutional affiliations.

## Supplementary Material

Supplementary Information
