# Supplementary material for: Biosynthesis of magnetic nanoparticles by human mesenchymal stem cells following transfection with the magnetotactic bacterial gene mms6
Source: Sci Rep. 2017 Jan 4;7:39755. doi: 10.1038/srep39755 (PMC5209691; doi:10.1038/srep39755)
Supplement: Supplementary Information [file srep39755-s1.pdf]

Biosynthesis of magnetic nanoparticles by human mesenchymal stem cells following transfection with the magnetotactic bacterial gene mms6.

Alistair Elfick[1,2],
Grigore Rischitor[3],
Rabah Mouras[1,8],
Asim Azfer[3],
Lisa Lungaro[1,3],
Marc Uhlarz[4],
Thomas Herrmannsdörfer[4],
John Lucocq[5],
Wesam Gamal[6]
Pierre Bagnaninchi[6],
Scott Semple[7],
Donald M Salter[3]

[1] University of Edinburgh, Institute for Bioengineering, School of Engineering, Edinburgh, EH9 3FB, UK
[2] University of Edinburgh, UK Centre for Mammalian Synthetic Biology, Edinburgh, EH9 3FB, UK
[3] University of Edinburgh, Centre for Genomics and Experimental Medicine, MRC IGMM, Edinburgh, EH4 2XU, UK
[4] Helmholtz-Zentrum Dresden-Rossendorf, Dresden High Magnetic Field Laboratory (HLD-EMFL), Dresden, 01328, Germany
[5] University of St Andrews, School of Medicine, St Andrews, KY16 9TF, UK
[6] University of Edinburgh, Centre for Regenerative Medicine, Edinburgh, EH16 4UU, UK
[7] University of Edinburgh, Centre for Cardiovascular Science, Edinburgh, EH16 4TJ UK
[8]Current address: University of Limerick Department of Physics & Energy, Materials & Surface Science Institute (MSSI), Limerick, Ireland

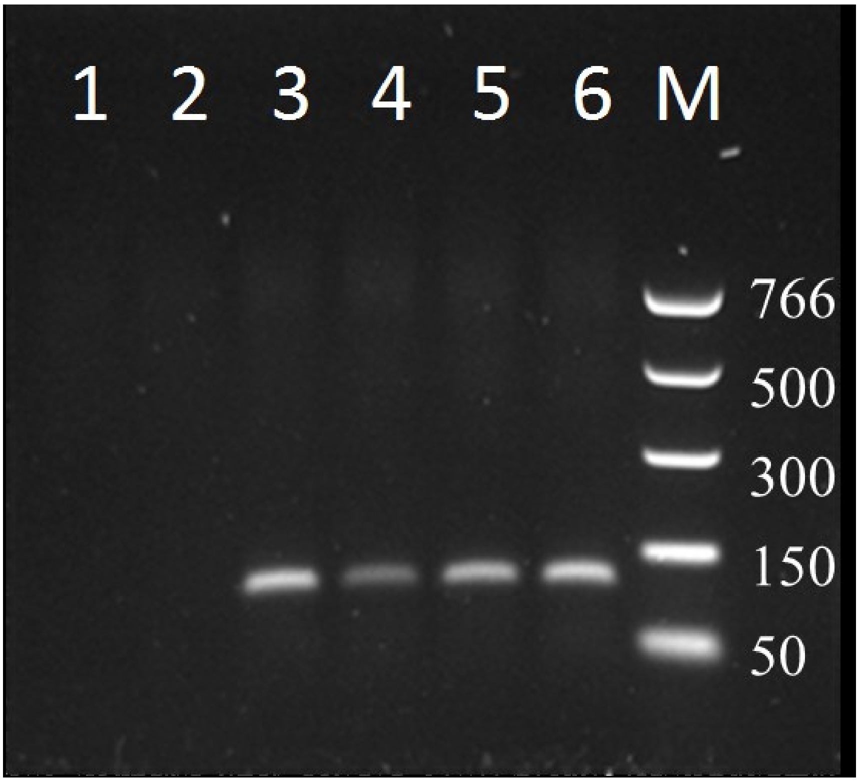

Supplementary Figure 1. Expression of the synthetic *mms6* gene by human MSCs.

1) Untransfected MSCs, 2) MSCs transfected with empty pcDNA3.1 vector 3) *mms6*-pcDNA3.1 vector, 4) MSCs transfected with *mms6* construct - day10, 5) MSCs transfected with *mms6* construct day 15 and 6) MSCs transfected with *mms6* construct day 21. M = DNA ladder (bp). Internal primer pair Forward: GTGGGGCCCGATTATTCT, Reverse: TCACGCAGTTCCACTTCTTC. Amplicon product length = 108 bp

A

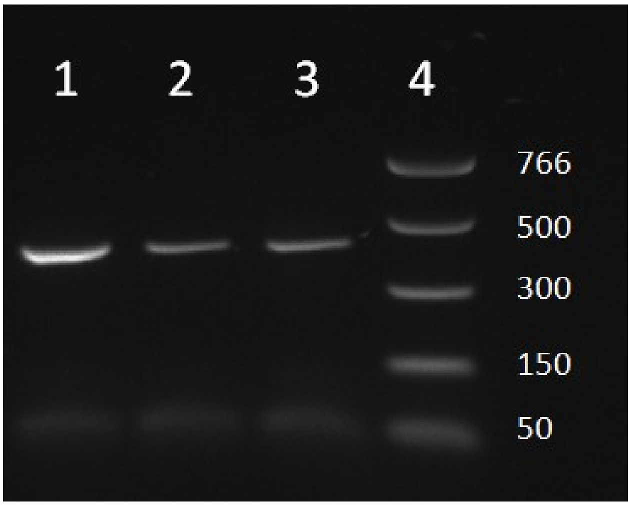

B

```
Ref.mms6:      ATGGGCGAAATGGAACGTGAAGGCGCGGCGGCGAAAGCGGGCGCGGCGAAAACCGGCGCG
PCR Day10      ATGGGCGAAATGGAACGTGAAGGCGCGGCGGCGAAAGCGGGCGCGGCGAAAACCGGCGCG
PCR Day15      ATGGGCGAAATGGAACGTGAAGGCGCGGCGGCGAAAGCGGGCGCGGCGAAAACCGGCGCG
PCR Day21      ATGGGCGAAATGGAACGTGAAGGCGCGGCGGCGAAAGCGGGCGCGGCGAAAACCGGCGCG
               ************************************************************

Ref.mms6       GCGAAAACCGGCGCGGTGGCGAAAACCGGCATTGCGGCGAAAACCGGCGTGGCGACCGCG
PCR Day10      GCGAAAACCGGCGCGGTGGCGAAAACCGGCATTGCGGCGAAAACCGGCGTGGCGACCGCG
PCR Day15      GCGAAAACCGGCGCGGTGGCGAAAACCGGCATTGCGGCGAAAACCGGCGTGGCGACCGCG
PCR Day21      GCGAAAACCGGCGCGGTGGCGAAAACCGGCATTGCGGCGAAAACCGGCGTGGCGACCGCG
               ************************************************************

Ref.mms6       GTGGCGGCGCCGGCGGCGCCGGCGAACGTGGCGGCGGCGCAGGGCGCGGGCACCAAAGTG
PCR Day10      GTGGCGGCGCCGGCGGCGCCGGCGAACGTGGCGGCGGCGCAGGGCGCGGGCACCAAAGTG
PCR Day15      GTGGCGGCGCCGGCGGCGCCGGCGAACGTGGCGGCGGCGCAGGGCGCGGGCACCAAAGTG
PCR Day21      GTGGCGGCGCCGGCGGCGCCGGCGAACGTGGCGGCGGCGCAGGGCGCGGGCACCAAAGTG
               ************************************************************

Ref.mms6       GCGCTGGGCGCGGGCAAAGCGGCGGCGGGCGCGAAAGTGGTGGGCGGCACCATTTGGACC
PCR Day10      GCGCTGGGCGCGGGCAAAGCGGCGGCGGGCGCGAAAGTGGTGGGCGGCACCATTTGGACC
PCR Day15      GCGCTGGGCGCGGGCAAAGCGGCGGCGGGCGCGAAAGTGGTGGGCGGCACCATTTGGACC
PCR Day21      GCGCTGGGCGCGGGCAAAGCGGCGGCGGGCGCGAAAGTGGTGGGCGGCACCATTTGGACC
               ************************************************************

Ref.mms6       GGCAAAGGCCTGGGCCTGGGCCTGGGCCTGGGCCTGGGCGCGTGGGGCCCGATTATTCTG
PCR Day10      GGCAAAGGCCTGGGCCTGGGCCTGGGCCTGGGCCTGGGCGCGTGGGGCCCGATTATTCTG
PCR Day15      GGCAAAGGCCTGGGCCTGGGCCTGGGCCTGGGCCTGGGCGCGTGGGGCCCGATTATTCTG
PCR Day21      GGCAAAGGCCTGGGCCTGGGCCTGGGCCTGGGCCTGGGCGCGTGGGGCCCGATTATTCTG
               ************************************************************

Ref.mms6       GGCGTGGTGGGCGCGGGCGCGGTGTATGCGTATATGAAAAGCCGTGATATTGAAAGCGCG
PCR Day10      GGCGTGGTGGGCGCGGGCGCGGTGTATGCGTATATGAAAAGCCGTGATATTGAAAGCGCG
PCR Day15      GGCGTGGTGGGCGCGGGCGCGGTGTATGCGTATATGAAAAGCCGTGATATTGAAAGCGCG
PCR Day21      GGCGTGGTGGGCGCGGGCGCGGTGTATGCGTATATGAAAAGCCGTGATATTGAAAGCGCG
               ************************************************************

Ref.mms6       CAGAGCGATGAAGAAGTGGAACTGCGTGATGCGCTGGCGCCACCAATG
PCR Day10      CAGAGCGATGAAGAAGTGGAACTGCGTGATGCGCTGGCGCCACCAATG
PCR Day15      CAGAGCGATGAAGAAGTGGAACTGCGTGATGCGCTGGCGCCACCAATG
PCR Day21      CAGAGCGATGAAGAAGTGGAACTGCGTGATGCGCTGGCGCCACCAATG
               ************************************************
```

Supplementary Figure 2. Expression (A) and sequence (B) of the synthetic *mms6* gene following transfection of MSCs and *in vitro* culture.

MSCs were transfected with the *mms6*-pcDNA3.1 vector and cultured for - lane 1) 10 days; lane 2) 15 days and lane 3) 21 days. Lane 4) = 766 bp markers. Following gel electrophoresis the PCR products were subjected to DNA Sanger sequencing and compared to the sequence of the initial synthetic *mms6* inserted within the pcDNA3.1 vector used for transfection. Sanger sequencing data confirmed 100% identity of the DNA sequence of the initial synthetic *mms6* gene and the gene expressed after 10, 15 and 21 days of transfection. CLUSTAL O (1.2.3) multiple sequence alignment compared with reference synthetic *mms6* gene sequence (Ref.*mms6*).

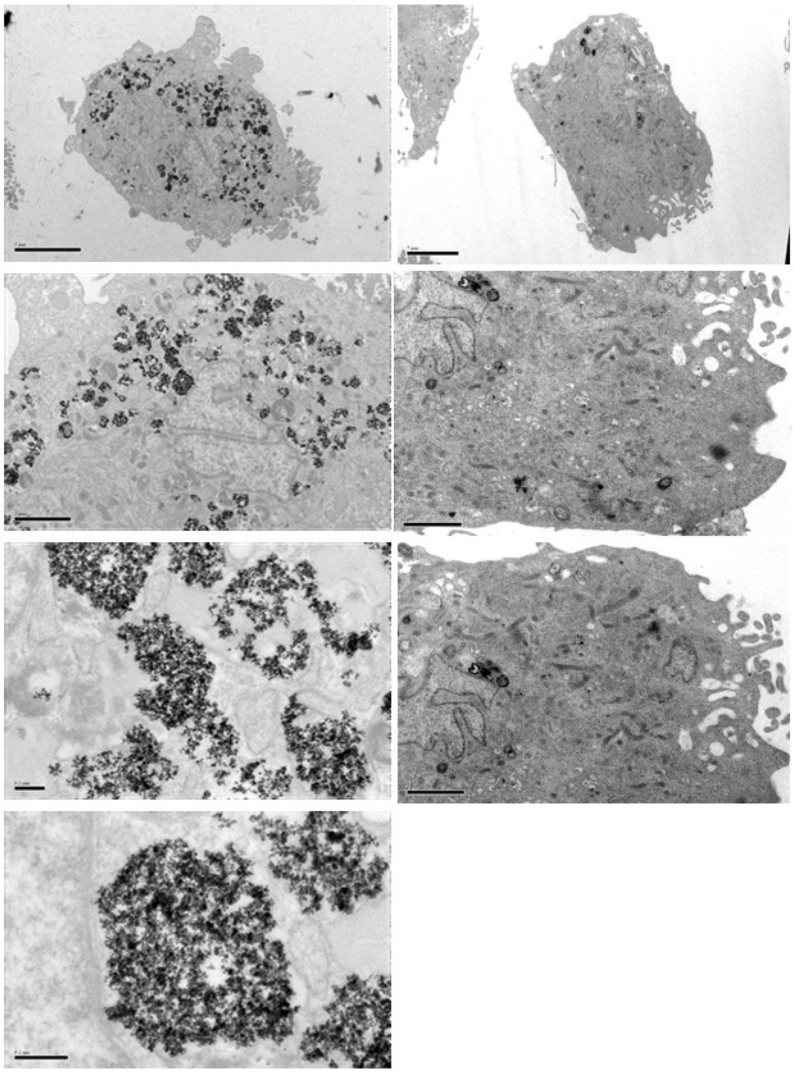

Supplementary Figure 3. Control TEM studies

TEM images of untransfected MSCs either loaded (left panel) or not loaded (right panel) with Fluid Mag DXS magnetic nanoparticles. Scale bars of images (from upper to lower): left panel - 5 µm, 2 µm, 0.2 µm, 0.2 µm; right panel 5 µm, 2 µm, 2 µm

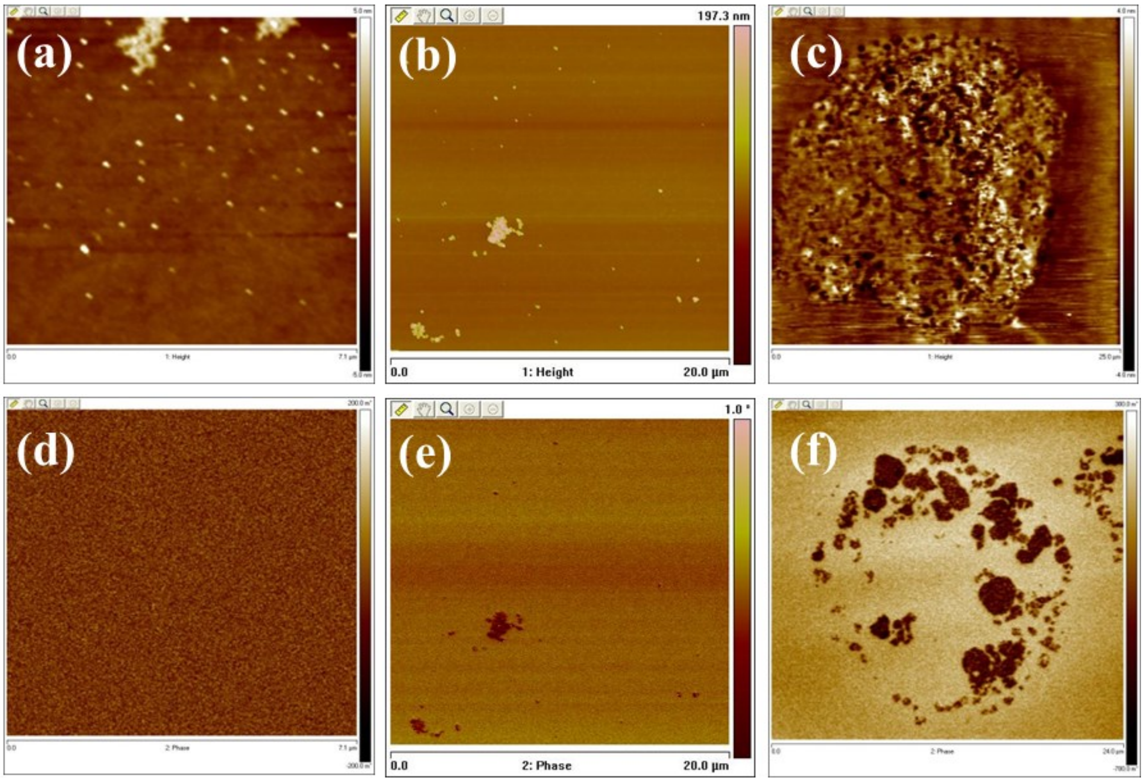

Supplementary Figure 4. Control AFM/MFM studies.
AFM / MFM of $FeCl_3$ powder (a, d): Fluid Mag DXS nanoparticles (b, e): MSCs incubated with Fluid Mag DXS (c, f). The upper row demonstrates topographic images whilst the lower demonstrates the equivalent MFM images. Magnetic particles are clearly identified in the MFM images as clusters of black spots due to attractive forces, showing the capability of MFM to detect magnetic paticles inside the cells. The absence of any features on (d) indicates the non-magnetic nature of $FeCl_3$ powder. Image widths: left panels 7.1 μm, centre 20 μm, right 25 μm (upper) 24 μm (lower).

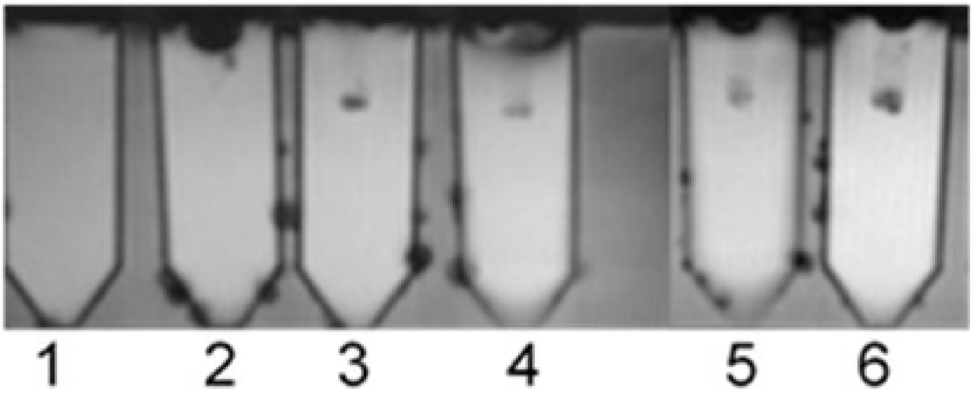

Supplementary Figure 5. MR images of Agarose Phantoms.

1. Agarose only, 2. 10^6 untransfected MSCs, 3. $2x10^4$ *mms6* transfected MSCs, 4. 10^5 *mms*6 transfected MSCs, 5. 10^6 *mms6* transfected MSCs, 6. 10^6 FluidMag DXS magnetic nanoparticle loaded MSCs

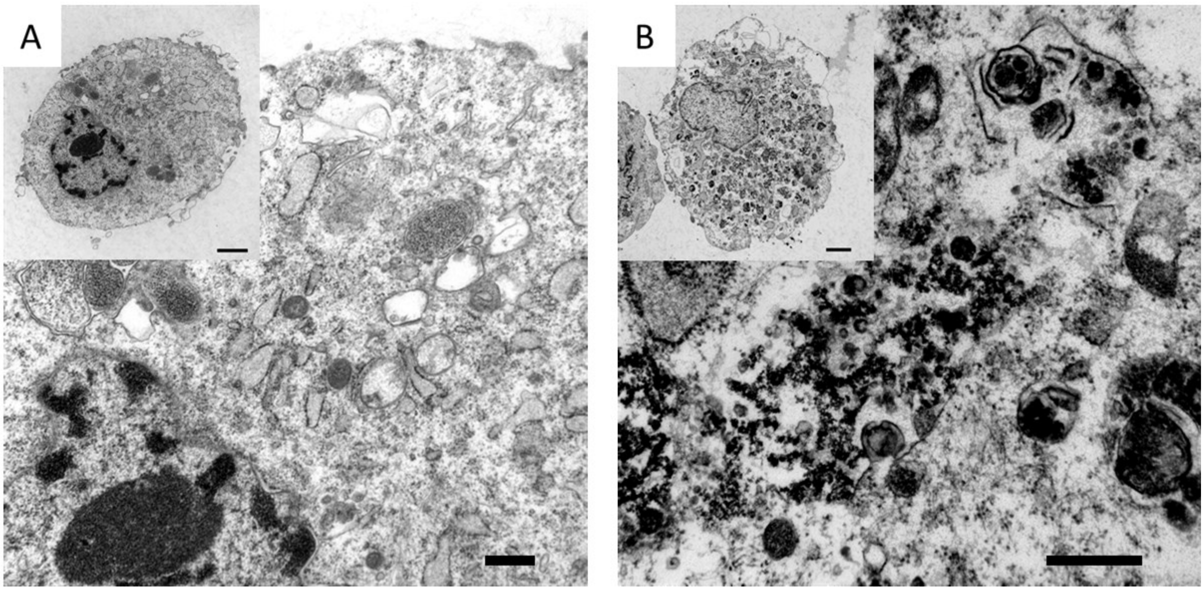

Supplementary Figure 6. TEM of *mmsF* transfected human embryonal bone marrow derived stem cells.

In contrast to untransfected MSCs (A), *mmsF* transfected MSCs (B) contain electron dense nanoparticles. MSCs were cultured in media containing 34mM ferric quinate. Bars in main figures = 500 nm; bars in inset = 2μm.

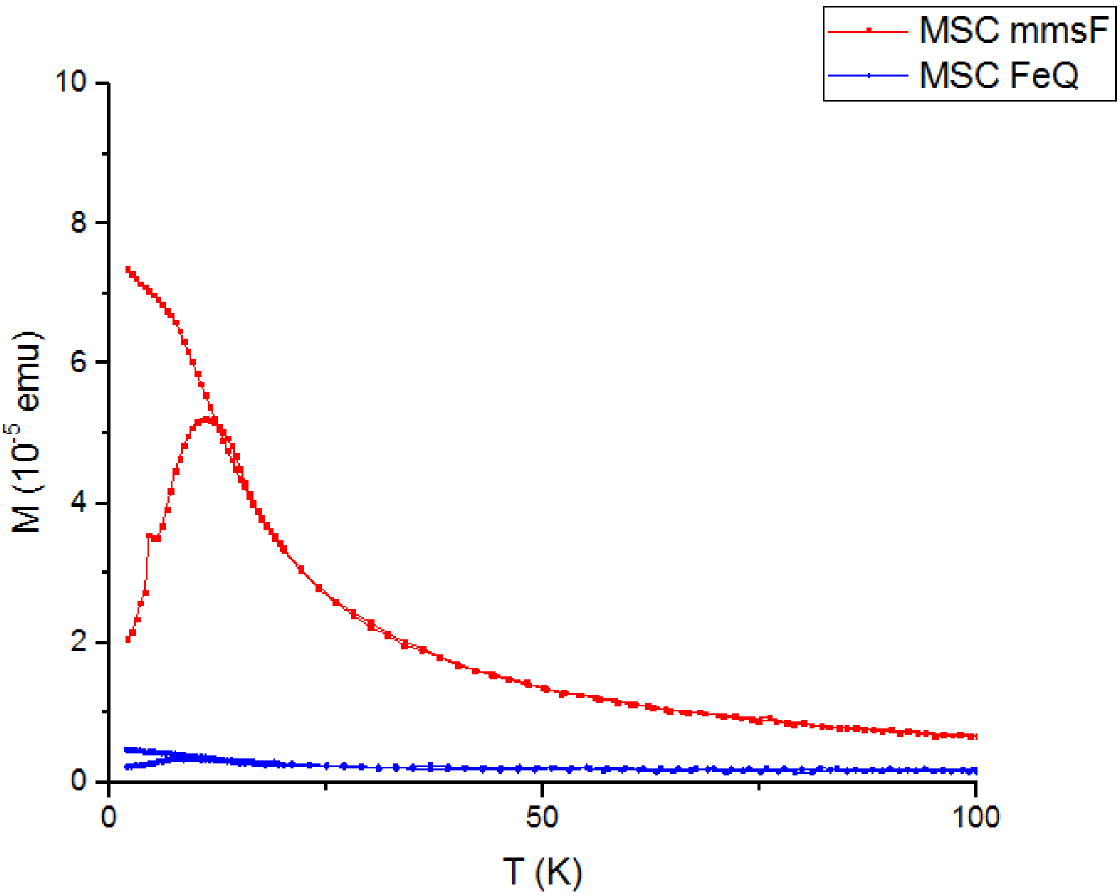

Supplementary Figure 7. SQUID Magnetometry of *mmsF* transfected human embryonal bone marrow derived stem cells.

mmsF transfected cells (MSC mmsF), cultured for 10 days, show magnetic behaviour as assessed by SQUID magnetometry whilst untransfected cells, cultured for 10 days in an identical 34mM ferric quinate containing media (MSC FeQ) show no magnetic behaviour.
